# Privacy-preserving breast cancer recurrence prediction based on homomorphic encryption and secure two party computation

**DOI:** 10.1371/journal.pone.0260681

**Published:** 2021-12-20

**Authors:** Yongha Son, Kyoohyung Han, Yong Seok Lee, Jonghan Yu, Young-Hyuck Im, Soo-Yong Shin

**Affiliations:** 1 Security Research Center, Samsung SDS, Seoul, South Korea; 2 Digital Health Business Team, Samsung SDS, Seoul, South Korea; 3 Division of Breast Surgery, Department of Surgery, Samsung Medical Center, Sungkyunkwan University School of Medicine, Seoul, South Korea; 4 Division of Hematology-Oncology, Department of Medicine, Samsung Medical Center, Sungkyunkwan University School of Medicine, Seoul, South Korea; 5 Department of Digital Health, SAIHST, Sungkyunkwan University, Seoul, Korea; 6 Center for Research Resource Standardization, Samsung Medical Center, Seoul, Korea; University College of Engineering Tindivanam, INDIA

## Abstract

Protecting patients’ privacy is one of the most important tasks when developing medical artificial intelligence models since medical data is the most sensitive personal data. To overcome this privacy protection issue, diverse privacy-preserving methods have been proposed. We proposed a novel method for privacy-preserving Gated Recurrent Unit (GRU) inference model using privacy enhancing technologies including homomorphic encryption and secure two party computation. The proposed privacy-preserving GRU inference model validated on breast cancer recurrence prediction with 13,117 patients’ medical data. Our method gives reliable prediction result (0.893 accuracy) compared to the normal GRU model (0.895 accuracy). Unlike other previous works, the experiment on real breast cancer data yields almost identical results for privacy-preserving and conventional cases. We also implement our algorithm to shows the realistic end-to-end encrypted breast cancer recurrence prediction.

## Introduction

There has been a rise in the security and privacy issues in many industrial fields, especially for medical applications, since medical data is considered to be personal and sensitive. There are growing legal restrictions regarding the transfer of personal data, including medical records, that restrict users from sending their data to such a platform to protect privacy. Meanwhile, thanks to rapid developments on deep learning field, it becomes possible to distil novel knowledge and build explanatory models from enormous amount of unrefined personal data. However those knowledge are also considered as a sort of sensitive data, it is highly restrictive to utilize them in real world. In short, huge amount of medical data accumulation provides new insights and novel methodology aided by deep learning, but the practical use of such academic progress is quite limited due to security and privacy issue at the same time.

As an elegant solution for this problem, both academic and industrial pieces of research contribute to apply privacy-enhancing technologies (PETs) such as homomorphic encryption (HE) and secure two party computation (2PC), which enables several parties to jointly perform computation on each own input data *while revealing nothing than the result*. Applied to deep learning, it enables several parties can jointly train a model while hiding each input data, or one party to obtain an inference result of the other party’s own model.

PET has been employed for privacy-preserving genome-wide association study (GWAS) [1, 2], medical (cloud) computation [3–6], and genomics diagnoses [7], and image processing by convolution neural network (CNN) [8–10] with remarkable performance improvements. However there are relatively less academic efforts on recurrent neural network (RNN) that plays an important role for time series prediction. Our work sheds a light on privacy-preserving inference of RNN, especially with a recently proposed gated recurrent unit (GRU) [11] model on breast cancer recurrence prediction model based on real world data.

### Related works

#### Privacy-preserving deep learning

Several studies solely rely on HE without any communication between the server and the client, except first and last ciphertext transmission. Deep neural network and CNN inferences have been actively studied from the seminal work CryptoNet [9] to the state-of-the-art Falcon [12], but it can only support a limited number of non-linear layers due to the homomorphic encryption limitation; Falcon shows 107 seconds latency for 3-layer CNN inference on CIFAR-10 [13] dataset, but the latency rapidly increases with layer size, and 7-layer CNN inference takes 1565 seconds.

Other studies overcome such scalability issue by combining HE with multi party computation, with a representative example GAZELLE [8]. This framework has been continuously improved so that the state-of-the-art CrypTFlow2 [10] can complete an inference of the practical CNN models like SqueezeNet [14] in less than 60 s and ResNet50 [15] in 10 min. However we note that it is difficult to find research related to RNN or Gated Recurrent Unit (GRU) due to the depth and complexity of the structure. To the best of our knowledge, there is only one published result on the privacy-preserving inference of RNN and GRU, PrivGRU [16]. This work exploits only secure two party computation, especially additive share [17], and shows approximately 30 s latency for one inference on the IMDb dataset [18] processed with 80 time steps. However, their experiment is executed in only one machine rather than communicating in a real network. Further, even the machine is equipped with GPU.

There are several works related to privacy-preserving authentication [19, 20], or privacy-preserving search on DB [21], but those works cannot allow computation on data in privacy-preserving sense. Finally, Vizitiu et al. [6] applied privacy-preserving deep learning to medical imaging (especially, X-tray coronary angiography view). They used an encryption scheme referred to as Matrix Operation for Randomization or Encryption (MORE), which does not guarantee the encrypted data’s security.

#### Prediction of time-to-event with censored medical data

Time-to-event (TTE) predictions are extensively used in clinical practices. Based on the studies conducted in the field of survival analysis using several algorithms, these can be divided into three different kinds: Statistical model, tree-based ensemble model, and deep learning-based model. The most popular statistical model is the Kaplan-Meier estimator, the non-parametric model [22] and semi-parametric Cox proportional hazard model [23]. Kaplan-Meier model and Cox model assume time-invariant effects of the covariates. The tree-based ensemble model is a random survival forest to avoid the proportional constraint of the Cox model [24]. The random survival forest is a tree-based method that constructs an ensemble estimate for the cumulative hazard function. Deep learning-based models are the most recent models such as DeepSurv [25], Cox-nnet [26], and RNN-SURV [27]. DeepSurv and Cox-nnet are based on the further development of the baseline Cox model with deep neural networks [25, 26]. Although these two models improved the prediction accuracy of TTE, time-varying covariates cannot predict survival curves. Owing to the sequential and time-based nature of the problem that frequently exists in clinical practice, RNN-SURV uses Long Short-Term Memory (LSTM) architecture and sequentially predicts a distribution over time to incorporate the patient’s longitudinal data [27].

#### Breast cancer recurrence prediction

Breast cancer is the most common, and the leading cause of cancer mortality among women in the world [28]. However, early detection of breast cancer by screening and advancing multidisciplinary treatment increases the survival rates of breast cancer patients.

Population-based survival, such as overall survival or disease-free survival, means the duration of survival after the disease was diagnosed or treated. However, the survival for each cancer patient can change over time in an actual situation. Therefore, there is a limitation in population-based survival, which does not reflect the change of many different factors that influence recurrence after diagnosis or treatment. There is a conditional survival analysis to overcome this limitation, which is the survival reflecting the disease-free time after diagnosis or treatment [29, 30]. However, conditional survival considers only a disease-free time, and this does not analyze several variable and considerable factors. This may affect the recurrence of breast cancer, such as blood test results, including white blood cell count, liver enzyme, tumor markers, etc., and radiologic results, including breast ultrasonography and mammography and so on.

Regarding this, a breast cancer recurrence prediction model using GRU is reported [31]. Here, we re-implemented the developed model by incorporating privacy-preserving methods and demonstrated the performance and usability of privacy-preserving data analysis by comparing the previous non-privacy model [31] and the proposed privacy-preserving method.

## Preliminaries

### RNN and GRU

**Fig 1:**
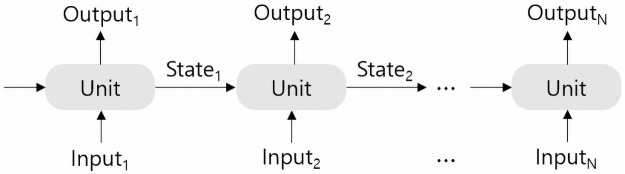
A RNN layer. Sequential RNN units.

RNN, a class of artificial neural networks developed to model sequential data such as time series or natural language. An RNN model takes the sequential input data and processes it through several layers, including the RNN layer. The RNN layer is composed with sequential RNN units as shown in Fig 1. There are several types of RNN units, such as a basic RNN unit, a well-known LSTM, and GRU [11], which we focus on in this study.

The GRU cell input comprises an input vector ${\vec{x}}_{t}$ of length *n*_*i*_ and a state vector ${\vec{h}}_{t-1}$ of length *n*_*h*_, where the initial state vector ${\vec{h}}_{0}$ is the zero vector. Then GRU cell computes the vector ${\vec{h}}_{t}$ that plays both output vector and state vector as following:

$$\begin{array}{ccc} {\vec{z}}_{t} & = & \sigma (W_{\vec{z}}{\vec{x}}_{t}+U_{\vec{z}}{\vec{h}}_{t-1}+{\vec{b}}_{\vec{z}})\\ {\vec{r}}_{t} & = & \sigma (W_{\vec{r}}{\vec{x}}_{t}+U_{\vec{r}}{\vec{h}}_{t-1}+{\vec{b}}_{\vec{r}})\\ {\vec{g}}_{t} & = & \text{tanh}(W_{\vec{g}}{\vec{x}}_{t}+U_{\vec{g}}{\vec{h}}_{t-1}⊙{\vec{r}}_{t}+{\vec{b}}_{\vec{g}})\\ {\vec{h}}_{t} & = & {\vec{z}}_{t}⊙{\vec{h}}_{t-1}+\left(1-{\vec{z}}_{t}\right)⊙{\vec{g}}_{t} \end{array}$$

where ⊙ is the component-wise vector multiplication, and *σ* is the sigmoid function defined by $\sigma \left(x\right)=\frac{1}{1+e^{-x}}$, and tanh is the hyperbolic tangent function defined by tanh(*x*) = 2 ⋅ *σ*(2*x*) − 1. The output state vector is fed into the next GRU cell. Every cell within the same layer uses the same weight matrices *W* and *U*, and bias vector $\vec{b}$, regardless of the index *t*. Hereafter, we call RNN layer composed with GRU cell by GRU layer, and RNN model that contains GRU layer by GRU model.

### Privacy-preserving inference scenario

The overall scenario and threat model resemble the previously performed studies on privacy-preserving inference [8]. Precisely, two parties are considered, the server and the client, where the server has a GRU model, mainly weight matrices and vectors, and the client has an input for the GRU model.

We assume that the architecture of the GRU model, including the number of layers, size of each layer, and the activation function for each layer, is shared between two parties, and the server retains the model parameters such as model matrix weights *W* and *U*, and bias vectors $\vec{b}$.

Our goal is to design a secure protocol between the server and the client that leads to the client obtaining the result of GRU model evaluation of its input, while no information is obtained by the the server regarding the input; furthermore, the client does not learn any more information about the GRU model than the information derived from the inference result (and the previously shared architecture). The security of our protocol is only guaranteed on semi-honest corruptions. This means that the server and the client faithfully execute the protocol without any malicious attempt, but only attempt to speculate the other party’s secret information.

### Homomorphic encryption

Homomorphic encryption (HE) refers to an encryption method that enables one to perform arithmetic between ciphertexts without decrypting them. In the recent past, various HE schemes [32–36] proposed different plaintext shapes and operations. In this study, we primarily use CKKS scheme [35] that supports encryption of real vectors and real arithmetic operations between each ciphertext. In particular, it typically supports component-wise addition and multiplication between ciphertexts, that is, SIMD (Single Instruction Multiple Data) addition and multiplication. For this reason, linear operations such as matrix multiplications match well with HE as reported in several works [37, 38].

Notably, there are limitations on the maximal length of vectors and the number of operations from the freshly encrypted ciphertext, according to bottom-level parameter selections. For HE operations transcending the limitations, one can use bootstrapping technique that restores operation capacity. Nonetheless, it is not a practical method yet. This feature makes non-interactive HE-only solutions to be narrowly practical for shallow-depth inferences.

Additionally, the HE scheme’s security is obtained from the complexity of challenging mathematical problems referred to as the Learning-with Errors problem [39].

### Secure two party computation

Secure Two Party Computation (2PC) enables two parties to jointly compute a function *f*(*x*, *y*) while allowing each party to retain its input in secret. There are various techniques for several input types and functions, and we mainly use Yao’s garbled circuit (GC) [40] that functions as represented by Boolean circuits. In other words, GC supports bit-string inputs $\vec{x}$ and $\vec{y}$, and bit-wise operation-based functions. For this reason, GC is more suitable for computations that a Boolean circuit can easily represent as a comparison of two inputs. Another well-known technique is Beaver’s technique [17]. It supports the addition and multiplication of two numbers, and hence it is more suitable for evaluating functions that are convenient to represent by the polynomial.

## Methods

This study was approved by the institutional review board (IRB) of Samsung Medical Center (IRB Approval No. 2020-06-026). The written consents were waived by the IRB since the data were analyzed anonymously.

### Data collection and description

Our work used the same dataset with the previous work [31], and here we provide some overview and refer details to [31]. We collected data from 13,117 patients diagnosed with breast cancer and who underwent breast cancer surgery at Samsung Medical Center (SMC) between 2000 and 2016. Of all populations, breast cancer resurfaced in 1,214 (9.2%) patients during the follow-up period. The cancer patients received regular tests during the follow-up period after surgery for surveillance. The median follow-up duration from the date of surgery to the last follow-up, including all-cause mortality, was 4.7 years, and the median number of visits was 8.4. In other words, they visited SMC twice a year on average. See Fig 1 in [31] for the inclusion and exclusion criteria of breast cancer patients in this study and the characteristics of the study population is shown in Table 1 of the reference [31]. We identified 31 prognosis features to develop a breast cancer recurrence model by feature selection analysis and clinician’s knowledge. The prognosis features used in the breast cancer model were 12 features related to the clinicopathologic category, four features related to treatment, and 15 follow-ups (time-dependent) features which refer to serial measurements during follow-up. The detailed chosen features are explained in the “Prognosis Feature Selection” section of [31] and more detailed information in the Supplementary Table 1 of [31].

Characteristic values were transformed to nominal or ordinal numeric ones. The stage and subtype were regenerated by considering hormone receptor status, tumor characteristics, and lymph node metastases. For continuous variables, log-transformation was used to deal with skewed data if needed, and Z-score normalization was applied. To reduce the number of discrete intervals of a continuous attribute, data binning divided the continuous feature (ki67) into a pre-specified number of categories (10% units); thus, making the data discrete. Categorical variables were one-hot encoded for the data analysis. As for the missing data, we used the average method for the data at the first time point and the LOCF (last observation carried forward) method for the data later.

### Our non-linear evaluation

**Fig 2:**
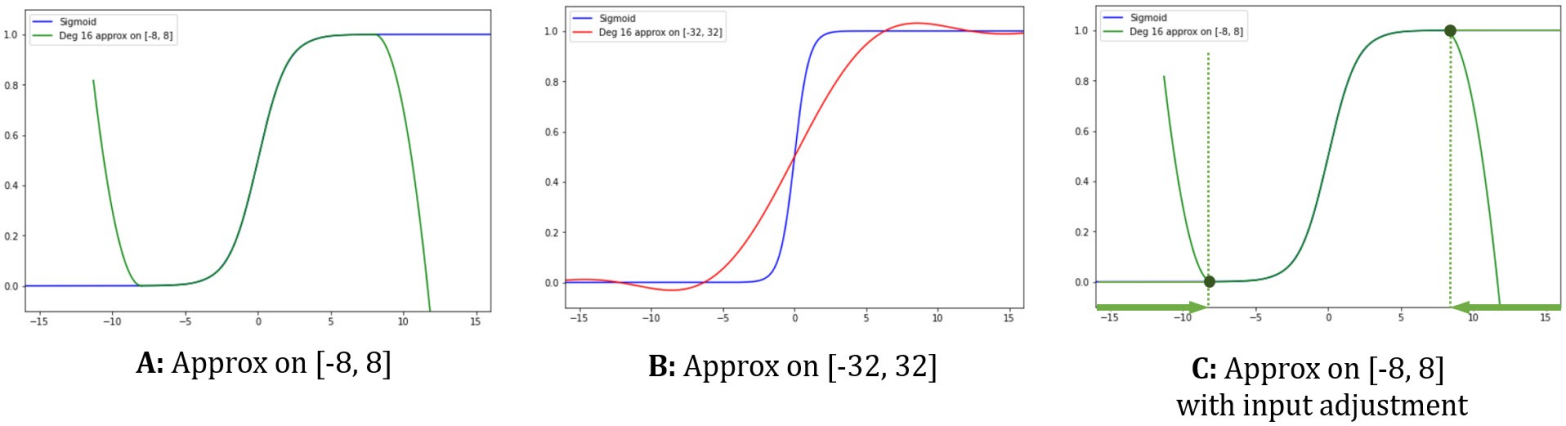
Evaluations of sigmoid with degree 16 approximations. A: Approx. on [−8, 8]. B: Approx. on [−32, 32]. C: Approx. on [−8, 8] with input substitution.

We briefly review why the non-linear part of GRU is challenging and present an overview of our approach. First, unlike the ReLU activation function of CNN, our target functions are not convenient to be represented by a circuit; further, 2PC-based evaluation is inefficient. The other prevalent way to evaluate non-linear function is to approximate it to proper polynomial and evaluate the polynomial by HE. However, this is also not satisfactory because of the following reason: As seen from Fig 2A, polynomial approximations rapidly diverge outside the approximation range. Thus, the inference result would be ruined if there is only a single input of activation function that deviates the approximation range during the entire inference procedure. Meanwhile, in our scenario, the server cannot see the input of the non-linear function because it is encrypted. Therefore, it has no choice but to consider a wide approximation range covering unexpected significant inputs of activation function. This problem is intensified for neural networks having deep structure, since it is hard to forecast the range input of activation functions. This results in a considerable decrease in approximation quality, leading to inaccurate inference result as Fig 2B shows.

To solve this, we observe that the GRU cell’s activation functions scarcely change outside some range. In other words, they rapidly converge to 1 and −1 outside some range. The evaluation result remains almost the same (with minor error) even if we substitute significant inputs with appropriate smaller values. Concretely, if the input is larger than 8, then substitute to 8 for *σ* evaluation. Therefore, if we add some procedure that adjusts the input of the server’s ciphertext in an encrypted state, then the server can approximate the target function to a much smaller range, and the total evaluation time is significantly reduced without sacrificing the quality of the results. Fig 2C shows our idea’s graphical explanation.

For the message adjustment, one may consider a simple protocol where the server sends the ciphertext of $\vec{m}$ so that the client directly performs comparisons in plain and return the ciphertext of the truncated message. However, this is undesirable for our threat model because the message $\vec{m}$ is a linear combination of the client’s input and the server’s model weights so that the client can learn model weights from simple linear algebra. In this regard, we design a secure HE-2PC hybrid protocol in the following section. An input HE ciphertext of a message is converted into an HE ciphertext—where the message is adjusted into some target range, without any information leak.

### Secure input adjustment

Assume that the server has a HE ciphertext of a real number *m* and the client has a HE decryption key sk. Our goal is to end with a HE ciphertext of $m_{i}^{\prime }$ for the server where

$$\begin{array}{c} m_{i}^{\prime }=\{\begin{array}{cc} R & \text{if}\;R<m_{i}\\ m_{i} & \text{if}\;-R\leq m_{i}\leq R\\ -R & \text{if}\;m_{i}<-R \end{array}, \end{array}$$

while either party cannot learn any information about $\vec{m}$ during protocol execution. Our protocol is described as follows:

- Step 1. the server converts a HE ciphertext of *m* into an additive share of *m*. Precisely, the server samples a random masking *r*, and using HE addition to obtain an HE ciphertext of *m* + *r*. It then sends it to the client, which decrypts the ciphertext to obtain *m* + *r* in plain. Then the server and the client respectively set each’s additive shares of *m* by *m*_*s*_ = −*r* and *m*_*c*_ = *m* + *r*.
- Step 2. Two parties execute 2PC protocol with each’s additive share, and the client obtains intermediate results required to compute *m*′. An immediate approach is to build 2PC to output Boolean bits *c*_*g*_ = (*m* > *R*) and *c*_*l*_ = (*m* < *R*), but letting the client directly know the comparison results may be perceived as a breach with regard to *m*. Instead, we let the server prepares two additional random bits *b*_*g*_ and *b*_*l*_ as inputs, and let the output of 2PC be two bits *h*_*g*_ ≔ *c*_*g*_ + *b*_*g*_ and *h*_*l*_ ≔ *c*_*l*_ + *b*_*l*_ so that the client is not aware of the comparison results. Finally the client encrypts *h*_*g*_ and *h*_*l*_ into HE ciphertexts and sends to the server.
- Step 3. the server recovers the final result from HE operations. It first compute ciphertexts of comparison bits by computing *c*_*g*_ = *h*_*g*_ − *b*_*g*_ and *c*_*l*_ = *h*_*l*_ − *b*_*l*_. Our resulting *m*′ can be represented by the following, and the server obtains the final ciphertext by homomorphically evaluating it:
  $$\begin{array}{c} m^{\prime }=\left(1-c_{g}-c_{l}\right)\cdot m+R\cdot \left(c_{g}-c_{l}\right) \end{array}$$

We finally remark an essential point; we take the integer part of *m*_*s*_ and *m*_*c*_, which introduces rounding errors of at most 1/2 for each input. This may lead to two undesirable outcomes:

- *m*′ remains *m* even when *R* < |*m*| ≤ *R* + 1,
- *m*′ is substituted by *R* (or −*R*) even when *R* − 1 ≤ |*m*| ≤ *R*.

Therefore, by considering the latter case first, the comparison target *R* has to be chosen so that *f*(*R* − 1) (rather than *f*(*R*)) is sufficiently close to 1, and to handle the former case, we have to approximate the target activation function *f* on [−*R* − 1, *R* + 1] (rather than [−*R*, *R*]).

### Secure GRU cell evaluation

**Fig 3:**
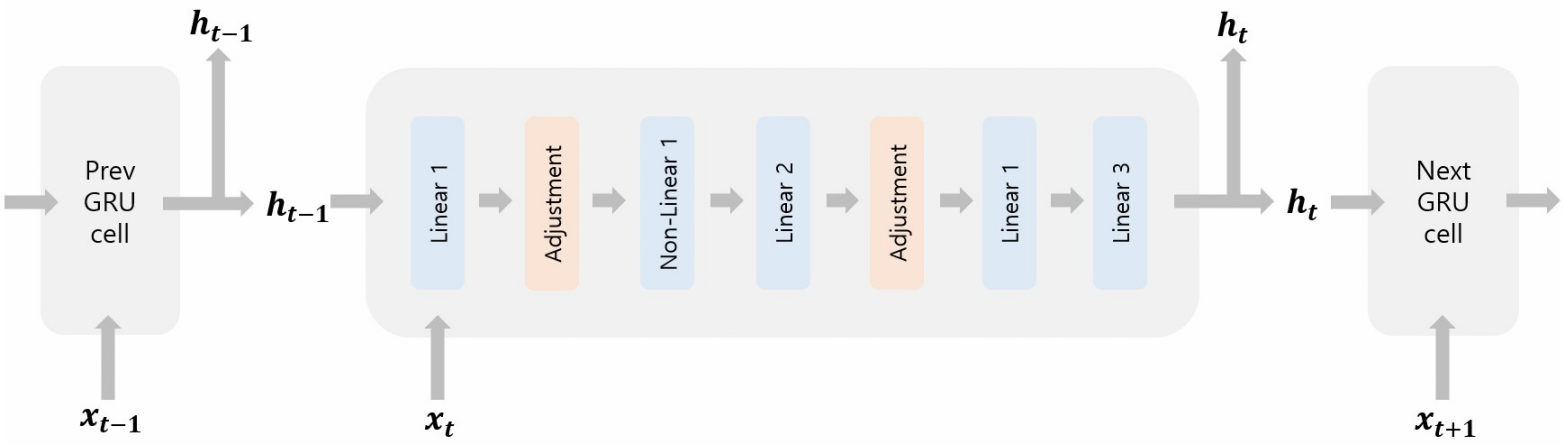
Workflow of GRU layer evaluation.

At this point, we are ready to describe an entire procedure of our GRU cell evaluation with a graphical workflow Fig 3.

- Linear 1 (HE): Compute ${\vec{z}}_{input}=W_{\vec{z}}{\vec{x}}_{t}+U_{\vec{z}}{\vec{h}}_{t-1}+{\vec{b}}_{\vec{z}}$, and ${\vec{r}}_{input}=W_{\vec{r}}{\vec{x}}_{t}+U_{\vec{r}}{\vec{h}}_{t-1}+{\vec{b}}_{\vec{z}}$.
- Input Adjustment (2PC): Adjust ${\vec{z}}_{input}$ and ${\vec{r}}_{input}$ to have small components.
- Non-linear 1 (HE): Compute ${\vec{z}}_{t}=\sigma \left({\vec{z}}_{input}\right)$ and ${\vec{r}}_{t}=\sigma \left({\vec{r}}_{input}\right)$.
- Linear 2 (HE): Compute ${\vec{h}}_{t-1}⊙{\vec{r}}_{t}$, and then ${\vec{g}}_{input}=W_{\vec{g}}{\vec{x}}_{t}+U_{\vec{g}}({\vec{h}}_{t-1}⊙{\vec{r}}_{t})+{\vec{b}}_{\vec{g}}$.
- Input Adjustment (2PC): Adjust ${\vec{g}}_{input}$ to have small components.
- Non-linear 2 (HE): Compute ${\vec{g}}_{t}=\text{tanh}\left({\vec{g}}_{input}\right)$
- Linear 3 (HE): Compute ${\vec{z}}_{t}⊙\left({\vec{h}}_{t-1}-{\vec{g}}_{t}\right)$ and then ${\vec{h}}_{t}={\vec{g}}_{t}+{\vec{z}}_{t}⊙\left({\vec{h}}_{t-1}-{\vec{g}}_{t}\right)$

We provide short explanations for matrix multiplications and approximation method, as given below. Particularly for matrix multiplications, we can use two different methods according to whether the client has a single inference query or sufficiently many inference queries that can be sent at once.

#### Single query

If the client has only one inference query, we have several ciphertexts that encrypt ${\vec{x}}_{t}$ for each time-step *t*. Then we have to perform matrix-vector multiplications, where matrices would be weight matrices *W* and *U* and vectors would be the input vector $\vec{x}$ and the state vector $\vec{h}$. There are many known methods for this, and we follow the naive approach of GAZELLE [8].

#### Batch query

For many real applications of RNN, an input vector $\vec{x}$ and a state vector $\vec{h}$ have a much shorter length than the typical choice of HE slot size *n*. Then if the client has sufficiently many queries for inference, it can improve throughput by encrypting a matrix *X* to fully exploit the slot size *n* where each column of *X* corresponds to several inputs (for the same time step). In this case, we have to compute matrix-matrix multiplication by HE, and we adapt the method of [38]. Notably, although the running time of 2PC grows almost linearly with the number of queries, the growth of HE computation is highly sub-linear to the number of queries, enabling us to achieve high throughput. Concrete experimental result can be found in Table 1.

**Table 1:** Benchmarks for one privacy-preserving GRU cell evaluations.

		Linear		Non-linear		Input Adjustment	
		Latency	Comm.	Latency	Comm.	Latency	Comm.
Input: 70 Output: 32	Single query	91	-	338	3.22	50	2.50
	32-Batch query	245	-	338	3.22	977	27.69
Input: 32 Output: 20	Single query	48	-	338	3.22	40	2.19
	32-Batch query	245	-	338	3.22	632	17.94

Runtimes are in milliseconds and comm. in MB

#### Approximation method

We approximate non-linear activation function to compute it by HE. In particular, we use the *Chebyshev* interpolant polynomial, which is reported to be suitable for HE computation [41, 42] is used. The detailed computation is also adapted from the same works [41, 42]. Precisely, we choose 24-degree Chebyshev approximation of *σ* over the range [−10, 10]. This approximation can also be used for tanh since tanh(*x*) = 2*σ*(2*x*) − 1 holds.

#### Bootstrapping HE with communication

Evaluation of all above HE parts in the server side alone requires large CKKS parameters, primarily owing to approximated polynomial evaluation. We instead use relatively smaller parameters and perform additional communication where the server sends the exhausted ciphertexts with random masking (to hide the intermediate computation result), and the client sends back the refreshed ciphertext by simple decryption-then-encryption. Finally, the server gets rid of the masking from the ciphertext and performs further HE operations.

### Model construction

To obtain a GRU model, we encode every non-numerical feature to have numerical vector inputs, and this results in a 70-length real vector for each exam record.

**Fig 4:**
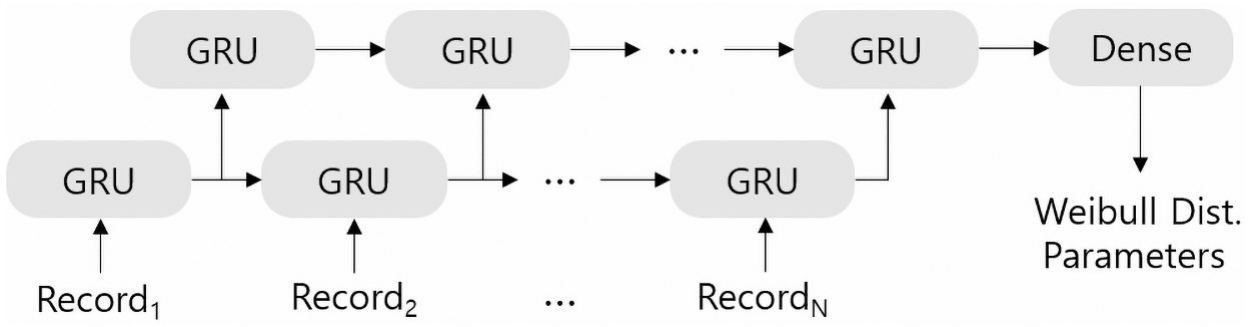
Our GRU model for predicting breast cancer recurrence.

We first rearrange the time-series sample data by collecting all exam records during the past 24 months for each patient. We have an *N* by 70 input sample corresponding to a patient with *N* exam records. Then we construct a GRU model as Fig 4, whose methodology is based on [43]: The first GRU layer is given time-series data of length 70 vectors and outputs again time-series data of length 32, and the second GRU layer only outputs the last hidden state, a vector of length 20. The final dense layer outputs two parameters for *Weibull distribution* so that one can draw a survival curve from these parameters to predict the expected survival time. For concrete implementation we use a python open-source machine learning library keras.

### Privacy-preserving inference

We obtain the entire privacy-preserving inference procedure of our GRU model by applying our GRU cell evaluation protocol. For this, the client the client encrypts each record vector into separate ciphertexts and sends them to the server. This lets the server know the number of visits of the corresponding sample from the number of ciphertexts. However, this is not considered privacy leakage in our case. Then the server evaluate two GRU layers according to Section. The final dense layer that consists of matrix multiplication followed by Weibull activation function evaluation remains to be explained. We note that, since the Weibull functions are one-to-one correspondences, providing the client the input of the final activation function is precisely the same as providing the final result in informational view and hence does not affect the security in any way. Thus, we let the server stop at matrix multiplication and allow the client to obtain the final results by performing the Weibull function evaluation in the plain.

## Results

We examine two PCs connected by a LAN network with bandwidth about 1Gbps. The server side PC is equipped with Intel Xeon E5-1650 running at 3.5 GHz, and the client-side PC is equipped with Intel Core i7-4790 running at 3.6 GHz. Our HE implementation is based on CKKS implementation of Microsoft SEAL library of version 3.5, and we use Yao’s garbled circuit implementation of MP-SPDZ [44]. Every parameter is chosen so that our protocol satisfies at least 128 bit-security.

We present some benchmarks for one privacy-preserving GRU cell evaluation in Table 1. The ‘Single query’ row and ‘Batch query’ row correspond to cases where the client only sends one inference query and several queries at once, respectively. For batch query case, we assume that the client sends 32 inference queries at once, where 32 is obtained from our parameter choice of CKKS scheme and the input vector length. To provide more information for HE-familiar readers, we use CKKS parameters that support *n* = 4096 length real vectors, and our input vector has length 70 for each time step. For efficient matrix multiplications, input vectors should be padded with zeroes to be power-of-two lengths 128. Finally, we can encrypt 32 = 4096/128 numbers of samples in one ciphertext.

We proceed to a full privacy-preserving inference of our GRU model for breast cancer recurrence prediction model, whose result from our dataset is presented in Table 2. Recall that our GRU model consists of two GRU layers and one final dense layer. For a sample having *N* exam records, we have to evaluate 2*N* consecutive GRU cells for two GRU layers, and one matrix multiplication for the final dense layer occupying a negligible portion in the total costs. Thus the total cost is 2*N* times of one GRU cell evaluation cost with a small additional cost for encryption and decryption. We emphasize that if the data owner insisting on privacy-preserving inference has sufficiently many queries so that sending more than 32 queries at once, it is definitively better to use a 32-batch query strategy that can process 139 inferences per one minute. Finally for accuracy comparison, we compute an index named *concordance-index* (C-index) [45] that is widely used for survival analysis. We present both C-index obtained from our privacy-preserving inference and C-index obtained from plain keras script in ‘C-index’ column of Table 2. As expected, we have no loss in privacy-preserving inference than plain inference results.

**Table 2:** Performance of privacy-preserving inference of our cancer prediction model.

	Encryption		Inference		Decryption		Total		C-index (Plain)
	Latency	Comm.	Latency	Comm.	Latency	Comm.	Latency	Comm.	
Single query	57	0.16	4889	57.10	< 1	0.08	4947	57.34	0.893 (0.895)
32-Batch query	57	0.16	13800	267.12	< 1	0.08	13858	267.36	

Runtimes are in milliseconds and comm. in MB. The inference costs heavily depends on the number of time records, and table represents costs for average of the number of records (about 5.13). The accuracy measured by C-index remains almost same with plain inference (Plain).

## Discussion

As medical data are the most sensitive personal data, there are many regulations to protect patients’ privacy. Additionally, there are growing concerns about a privacy breach. However, to develop new drugs, medical devices, or intelligence clinical decision support systems, medical big data analysis is essential. Diverse privacy-preserving techniques have been proposed to balance privacy protection and data usage. In this work, we proved the possibility of privacy-preserving inference on the real-time series medical data. To the best of our knowledge, this is the first study to validate the performance of privacy-preserving inference using actual patient data.

We demonstrated that our proposed method successfully predicted breast cancer recurrence with the normal inference model’s comparable accuracy. In the aspects of theory and implementation, we proposed a novel approach to combine privacy-enhancing technologies (PETs) such as homomorphic encryption (HE) and secure two party computation (2PC), which does not change the well-known activation function. Through the proposed model, we implemented privacy-preserving inference with the GRU-based model without degrading the performance. First, we propose an efficient protocol for privacy-preserving GRU evaluation by combining two privacy-enhancing technologies; HE and 2PC. Second, in our protocol, the model provider can use the original GRU model rather than training another (semi-)GRU model tailored for PET-friendly computations. Our protocol also performs almost the same computation as the plain one, and hence privacy-preserving GRU inference service based on our protocol implies no accuracy loss. Lastly, we also implement our protocol and apply it to real-world data to predict breast cancer recurrence.

We finally note that, although batch query cases can also be applied to real scenarios (e.g., inference query from a large hospital’s database), one inference query case is indeed a natural approach for privacy-preserving inference for future research. Thus acceleration of one inference query case by improving our protocol or applying another PET would be an interesting direction in future.
